# Understanding a population: A methodology for a population-based coastal safety survey

**DOI:** 10.1371/journal.pone.0256202

**Published:** 2021-08-13

**Authors:** Jasmin C. Lawes, Lea Uebelhoer, William Koon, Luke Strasiotto, Frederic Anne, Shane Daw, Robert W. Brander, Nick Mulcahy, Amy E. Peden

**Affiliations:** 1 Surf Life Saving Australia, Sydney, New South Wales, Australia; 2 School of Biological Earth and Environmental Sciences, UNSW Sydney, Sydney, New South Wales, Australia; 3 Beach Safety Research Group, UNSW Sydney, Sydney, New South Wales, Australia; 4 OmniPoll Market Research, Sydney, New South Wales, Australia; 5 Surf Life Saving New South Wales, Sydney, New South Wales, Australia; 6 School of Population Health, UNSW Sydney, Sydney, New South Wales, Australia; Bucharest University of Economic Studies, ROMANIA

## Abstract

Drowning is a global public health problem, but accurately estimating drowning risk remains a challenge. Coastal drowning comprises a significant proportion of the drowning burden in Australia and is influenced by a range of behavioural factors (e.g. risk perception, knowledge, attitudes and behaviours) that are poorly understood. These factors, along with those that impact exposure (e.g. coastal visitation and activity participation) all impact on drowning risk. While excellent mortality and morbidity data exists in Australia, a lack of coastal participation data presents challenges to identifying high-risk groups or activities and prioritising prevention efforts. This methods paper describes the development and evolution of an ongoing, annual, nationally representative online survey as an effective tool used to capture valuable data about the Australian population’s relationship with the coast. This paper explores how the survey is structured (12–14 sections spanning multiple topics and themes), the different question types used (including open text, 4-digit responses and categorical questions), the sample size (1400–1600 respondents), sampling strategy (using demographic quota sampling which can then be post-weighted to the population if required) and how topics and themes have changed over time to enhance the quality of data collected (i.e., wording changes to enhance participant comprehension or data usability and changing issue-specific ‘feature’ topics of interest such as campaign evaluation). How the survey is implemented online is described, both practically through to third-party recruitment processes and ethically to maximise anonymity of respondents and ensure data quality. Interim analyses indicate the impact of considering exposure when calculating fatal drowning rates, especially by activity (e.g., crude boating drowning rate 0.12 per 100,000 population vs 0.95 per 100,000 exposed population [relative risk = 8.01; 95% confidence interval: 4.55–14.10]). This study highlights lessons learned in the process of conducting a nationally representative coastal participation survey as well as the strengths and limitations of adopting this approach. Data collected will provide more detailed information on the skills, behaviours, knowledge and attitudes of coastal activity participants. Analyses of this unique dataset will inform research that will underpin development and evaluation of coastal drowning prevention initiatives prioritising those most at risk. It is hoped that the methods detailed within this study may be useful for other countries to develop similar approaches to understanding their own population.

## Introduction

The World Health Organization (WHO) has described drowning as a threat to global public health [1] with estimates of the unintentional fatal drowning burden ranging from 320,000 (2016) to 236,000 in 2019 [2]. The Global Burden of Disease (GBD) study estimates 295,000 deaths from drowning globally in 2017, a rate of 4.00 per 100,000 people [3]. Coastal environments, such as beaches, oceans and rocky foreshores are locations that significantly contribute to the overall drowning burden [4]. In Australia, coastal waterways account for an average of 112 drowning deaths annually (a mortality rate of 0.45/100,000), with 125 fatalities recorded in the 2019/20 financial year [5]. While a relatively low rate of fatal coastal drowning is observed in Australia, coastal activity and participation are dynamic and thought to be increasing. Moreover, a significant number of non-drowning fatalities and injuries are associated with other causes, and more than 10,000 ocean rescues occur each year on the Australian coast [6, 7] with little evidence that suggests these numbers are improving. This highlights the need for a more focused effort to better understand the relationship(s) between the dynamic, highly variable, and often hazardous Australian coast and the people who interact with it.

Epidemiological studies have provided the majority of our understanding of who drowns in Australian coastal environments and the risk factors implicated in these fatalities [7–13]. However, coastal drowning risk is also influenced by a range of factors where evidence is scant, including individuals’ risk perception, knowledge, attitudes and behaviours, as well as factors that impact exposure such as coastal visitation and participation in various activities [8, 10, 11]. Some behavioural research into coastal drowning from Australia has explored beachgoer knowledge and attitudes towards safety [11] and the impact of signage and beach flags [10, 14], with a significant body of work focused on the rip current hazard [8, 10, 15–17]. However, studies with a national scope, or which are focused on individuals’ risk perception, alcohol use, lifejackets and the impact of weather on coastal drowning risk, as examples, are relatively rare. These areas represent important knowledge gaps and future work which addresses these gaps will significantly enhance understanding of the problem and inform best practice in lifeguarding and education [4].

Exposure to drowning risk remains poorly understood globally [18], including in the coastal environment [4]. Many epidemiological studies report rates of fatal drowning per head of resident population. While a more robust measure than counts alone, such rates are not entirely accurate as risk is underestimated if attributed to both exposed and unexposed groups. Few studies have explored the impact of exposure on drowning risk [19], including in the coastal environment [20–23], or while undertaking coastal activities such as rock fishing [24] or scuba diving [25]. The few peer-reviewed studies that have been conducted to date, collate data through direct observations from a small number of locations (i.e., 29 beaches in one Australian state [20], wave dominated beaches in one Australian state [21, 23]), on specific variables (i.e., in-water bather counts [20], exposure to water, duration and distance from shore during bathing episodes and beach visits [21, 23]), and over shorter time periods (e.g. data collected during the summer months only [20, 21]). Another health survey used computer assisted telephone interviewers (CATI) to ask respondent about exposure to water, type of aquatic location visited (swimming pool, beach, lake, river, creek, stream or dam) and selected activities (swimming, fishing or rock fishing), but this was across a single Australian state and included only the previous four weeks prior to the survey [24]. A previous study exploring exposure impacts on fatality rates among scuba divers, derived participation estimates using denominators from three separate surveys (national survey on participation in exercise, recreation and sport, surveys of international tourists in the state of Queensland and one dive operator in the state of Victoria [25], while this paper begins to address this knowledge gap for scuba diving, this has not been attempted for other popular coastal activities. Importantly, no study has explored this issue at a national level in Australia, or across multiple years and activities.

Many factors impact on drowning risk including who visits a location, how often they visit, how long they stay, which activities are undertaken, and which safety behaviours are observed [22–24, 26]. Visitation and activity participation are also influenced by temporal and environmental factors such as time of day, day of week, season and weather. Although various methods have been previously used, including direct observation [23, 26] and survey data [19, 22, 24], capturing data on the exposure to drowning risk is difficult, time consuming and expensive. The methods presented here will detail the development of a valuable database that will enable a more accurate estimation of exposure and risk to guide preventative efforts.

In order to capture valuable data about the Australian population at large in relation to their beach visitation, usage and knowledge of the coast, nationally representative surveys funded by Surf Life Saving Australia (SLSA) have been conducted online since 2014. SLSA is Australia’s peak coastal safety body and an iconic organisation that provides considerable benefits to the community through coastal safety and lifesaving services aimed to significantly reduce injuries and fatalities on our coast.

This study aims to explore the method used, the implementation of, and the lessons learned in the process of conducting this nationally representative coastal participation survey to inform drowning prevention efforts in Australia. It is hoped that this methods paper will help guide other countries in conducting similar surveys to improve their understanding of coastal participation and safety in their own population. This paper presents the methodology utilised in development and implementation of the survey, preliminary findings and reflect on the importance of this data for coastal drowning prevention initiatives, by discussing the lessons learned in this process.

## Methods

The following section outlines the survey context, development and implementation process including recruitment, incentives, quality assurance and survey structure. The question types used to explore topics and themes are also examined, including how they have developed and changed over time to enhance the survey as a whole and the quality of the data it collects.

### Survey development

#### Survey context

Prior to 2014, OmniPoll (a market research agency, then called Newspoll) conducted a general survey of the Australian public via an annual national telephone omnibus. In addition to questions on a variety of other topics, SLSA included questions in these surveys related to surf safety and rip currents, which are the primary hazard causing rescue and drowning on many Australian surf beaches [12, 27]. These questions primarily focused on Australians’ understanding of rip currents and their associated risks, but did not include other aspects of coastal risk and safety. To augment this existing effort, SLSA held a series of internal workshops which identified another significant data deficiency: the lack of information related to coastal exposure among the Australian population. While excellent mortality and morbidity data exists, this lack of coastal activity participation data presents serious challenges to identifying specific high-risk groups or activities and prioritising prevention efforts. The rate of injury provides a contextual measurement of mortality and morbidity relative to the population when compared with crude drowning numbers, which may mask key trends in behavioural change and the effectiveness of intervention strategies. To address this knowledge gap, a draft questionnaire was developed to include a range of topics such as participation in coastal activities, risk perception, swimming ability, and safety practices.

Building on from this draft questionnaire, SLSA engaged OmniPoll in 2014 to undertake the development and delivery of a new annual study specific to coastal safety (referred to as the SLSA National Coastal Safety Survey–NCSS), which was the first comprehensive national survey to explore how Australians use the coast, how they behave around the coast and their understanding and perception of the risks associated with the coast. Due to the size and scope of this study, an online survey was recommended as the best approach as it allowed for the inclusion of stimulus material, such as images or videos, and minimised the risk of social desirability bias [28]. OmniPoll recommended that the survey length necessary to meet the objectives was 15 minutes (although it is currently closer to 20 minutes with subsequent topic expansions) and the survey tool was amended to include all relevant demographics and coastal participants. Using the questions drafted by SLSA, OmniPoll developed a fully scripted questionnaire which was pre-tested and validated using cognitive testing. Cognitive testing is a standard process to validate a questionnaire and tests that respondents understand the survey questions well and as they are intended [29]. Cognitive testing of this initial questionnaire was conducted by OmniPoll staff face to face with six respondents who answered a paper version of the questionnaire, and their level of understanding was monitored concurrently. This was particularly important to ensure that the survey was relevant across people of different ages (16–69 years at that time).

Since 2014, the NCSS has been conducted annually online. The scripted questionnaire is reviewed each year allowing for developments and improvements and the addition of subject-specific sections where a particular topic can be featured (e.g. alcohol/drug use and coastal recreation, marine stingers, lifejacket perception and behaviour). The survey is conducted at the same time each year (April–autumn in Australia), with largely the same questions in relatively the same order (options within overarching questions are presented randomly to avoid ‘order effects bias’ [30]). While the survey has continued to grow and develop, the questions (topics and themes), structure and wording have remained relatively consistent, with approximately 80% of questions being asked consistently each year.

### Participant recruitment

#### Survey delivery and demographic quota sampling methodology

Each year, participants are recruited via a third-party market research online panel company (Lightspeed, [31]). Lightspeed sends recruitment invitations to panel members via email, with a link to access the online survey via their portal. Once participants have logged into their Lightspeed member portal, they actively choose to participate by clicking another link to access the online survey. This process provides a dual phase opt-in strategy, which increases survey uptake by committed participants who are more likely to fully complete the survey. Initial invitations are emailed to panel members based on pre-determined demographic quotas, which can then be post-weighted to Australian population data to ensure a representative sample of the Australian population. A follow-up reminder email is distributed weekly to engage panel members in the same manner as the initial contact. The number of reminders a person receives is dependent upon the demographic quota the potential respondent fits within, i.e. harder to fill quotas get more reminders. Once a quota has been achieved, the link is no longer available to panel members within that demographic. Contact strategies involve no pressure or coercion (real or perceived) to participate and ensure participation is voluntary. This is achieved though all lightspeed panellists having voluntarily joined Lightspeed membership and then have decided to complete this survey. Market research is conducted within its own ethical guidelines, and approved policies and procedures. OmniPoll and Lightspeed operate under the Australian Privacy Principles contained in the Privacy Act 1988 [32]. Both organisations also adhere to the Privacy (Market and Social Research) Code 2021 [33]. This survey has been conducted within these approved market research procedures.

Surveys can be completed on laptops and mobile devices. Surveys can be completed on laptops and mobile devices. No pre-screening process is required for participants aged 18 years and above who qualify for either the entire survey or a component of the survey. An exception, however, is for children aged 16 and 17 years, who require parental consent prior to participation. Children (aged 16 and 17 years) must be logged into the survey portal by a parent, who is then required to provide consent prior to survey access. Once a potential participant is determined to be eligible to participate and has indicated their interest in participating, they provide consent and commences the survey. Participants are informed prior to undertaking the survey that the data will be used for external research, are given the option to opt out of the survey at any time and are given the option to learn more about the survey and why it was being undertaken after completing it. After a relative period of time has passed (an average of ten days) and at the discretion of the project manager (OmniPoll), the survey is closed and recruitment ceases. Similarly, demographic quotas are closed once they become full.

A cohort of survey participants is determined such that it is representative of the Australian population (resulting in approximately 1400–1600 survey respondents each year). The survey is provided to the participant without identifying SLSA to avoid any bias in answering questions either for or against SLSA. Lightspeed have a panel of approximately 200,000 people who undertake surveys, from this panel the NCSS is offered to people based on pre-determined, representative demographic quotas (including targeted age groups, state–metropolitan and regional, and gender). Specifically, the triple interlocking quota sampling method is used, using age by gender by regional area to determine demographic quotas. Each response can then be post-weighted to Australian Bureau of Statistics depending on the specific demographic they represent (using the pre-determined interlocking quota determined by age, sex and regional area), meaning that some responses are weighted to represent a value greater than one and others to less than one. For example, if the number of responses from a specific target demographic (e.g. 50–54 year old males in regional areas of the Australian state of Victoria) have not have achieved the intended population target, the received responses will be weighted at 1.1, in contrast to a demographic that may have received responses in excess of the intended population target (e.g. 20–24 year old males in metropolitan areas in the Australian state of New South Wales) and whose responses are then weighted at 0.9. This ensures the survey has an effective sample size for analysis (16–69 years old, approximate n = 1,400 for 2014–17; 16 years old and above, approximate n = 1,600 since 2018). These numbers are flexible and can be adapted to suit requirements. For example, in 2015 the sample size of the Australian state of Tasmania was doubled from 50 to 100 after concerns from Tasmanian state entities that Tasmanian behaviours were not being accurately represented. Similarly, in 2018, the age group 70 and over was added to the survey when internet penetration in this age group allowed for accurate collection of data (in 2015 internet penetrance was >90% for those aged 35 years and older [34]). Apart from these instances the data groups and representative samples have remained consistent over time, although we hope to be able to increase number of respondents in the future.

#### Remuneration and quality assurance

Survey participants receive financial remuneration for their participation by means of earning ’points’ (with a small associated financial value), which can then be reimbursed as monetary deposit or used to purchase vouchers. The incentive for this survey is up to an equivalent of AU$5. Prior to undertaking the survey, participants are informed of the average time taken to complete the survey and of the financial reward for completing the survey. The exact dollar value of the points awarded depends on the redemption option chosen by the participant. The reimbursement/reward is provided to participants at the completion of the survey to their Lightspeed account. The monetary value of the survey fluctuates for different users depending on what priority population they represent and the current need for them to contribute in order for the survey to reach national representation quota numbers. Due to the low population size of the Northern Territory, the survey is open to all respondents aged 16 and above and is only closed at the end of the survey to obtain a large enough sample.

There are multiple quality assurance assessments in place to ensure the surveys are being completed accurately and honestly. Participants are flagged by Lightspeed and no remuneration occurs if they are found to answer the survey dishonestly, demonstrate acquiescence bias or deliberately attempt to complete the survey as quickly as possible through nondifferentiation bias or “straight-lining” [35]. These responses are then excluded from the omnibus. Moreover, the survey includes hidden questions to direct respondents through the survey, for example to allocate respondents to activity sections for which they have participated in at least three times in the last year.

### Survey questions and themes

#### Question types

The questionnaire contains multiple question types, including open text, 4-digit responses and categorical questions (Table 1). Only one 4-digit response question is included each year and asks participants for their current residential postcode, while open text questions ask the respondents to write their answers in their own words. Categorical questions can be divided into multi-response, multi-response and other, single response, single response and other and single response grid questions. Multi-response questions (n = 141) allow the participant to select multiple response options while only one answer can be selected for single response questions (maximum available n = 309). The addition ‘and other’ allowed the respondent to either select a given response option or they could add their own response (multi + other, n = 42; single + other, n = 12). Single response grid questions (herein referred to as single-grid; n = 1200) consist of a main question with multiple subcomponents. For example, a main question might ask the participant about safety practices when swimming or wading, and the subcomponents specify different scenarios such as ‘swim or wade at a patrolled beach during patrol times’ or ‘swim or wade between the red and yellow flags.’ For each of these scenarios, the participant is asked to select one answer (*always*, *most of the time*, *sometimes*, *never*, *can’t say*) for each subcomponent. Single-grid is the most common question type (75% of all questions; Table 1).

**Table 1:** Question types included in the survey with example questions and responses, average numbers ($\bar{x}$) asked each year plus standard error to show variance.

Question Type	Description	Example of question	Example of response options
**Open text**	Respondents could write their answer in their own words.	Please indicate why you **do not always** look for the presence of rip currents in the area prior to entering the water? (*Please type in your responses in the box provided)*	Open text between 50–500 characters
$\bar{x}$ = 4.4 ± 0.83			
**Categorical–multi response (multi)**	Respondents were asked to select all answers that applied.	Into which of these age groups do the children in your household belong? (*Select all that apply)*	· 4 years or under
			· 5 to 9
$\bar{x}$ = 20.1 ± 1.02			
			· 10 to 12
			· 13 to 15
			· 16 to 17
**Categorical–multi response + other (multi and other)**	Respondents were asked to select all answers that applied and could also add their own response.	Where do you usually seek information regarding coastal safety? *(Select all that apply)*	· Online
			· TV
			· Radio
$\bar{x}$ = 6.0 ± 0.67			
			· Newspaper
			· Magazine
			· Regular email newsletter
			· Using an app on smartphone or tablet
			· Other (please specify)
			· None of these / Can’t say
**Categorical–single response (single)**	Respondents were only allowed to select one answer.	Thinking about rip currents that occur at surf beaches. How confident are you that you could identify a rip current? *(Select one answer)*	· Very confident
			· Somewhat confident
$\bar{x}$ = 44.1 ± 3.80			
			· Not very confident
			· Not at all confident
			· Can’t say
**Categorical–single response + other (single and other)**	Respondents were asked to select either a given response option, or they could add their own response.	How did you get out of the rip current? *(Select one answer)*	· I swam and managed to get out by myself
			· A surfer helped me / rescued me
$\bar{x}$ = 1.7 ± 0.69			
			· A lifesaver or a lifeguard helped me / rescued me
			· Someone else helped me
			· I floated with the current and it returned me to shore
			· Other (please specify)
			· Can’t say
**Categorical–single response grid (single-grid)**	The question consisted of a main question with different subcomponents. Respondents were asked to only select one answer for each subcomponent.	Please indicate how often you personally follow each of these practises when you go swimming or wading? *(Select one answer per row)*	· Always
			· Most of the time
			· Sometimes
$\bar{x}$ = 150.0 ± 16.69		· Swim or wade at a patrolled beach during patrol times	· Never
		· Swim or wade between the red and yellow flags when you are on a patrolled beach	· Can’t say
		· Swim or wade with at least one other person you know	
		· Check surf conditions with a lifesaver, lifeguard or other authoritative source	
		· Check for and obey safety signs posted on the beach	
		· Look for the presence of rip currents in the area prior to entering the water	
		· Avoid swimming or wading under the influence of alcohol \ drugs	
		· Follow the advice of the local lifesaver or lifeguard when you are on a patrolled beach	
**4-digit response (4-digit response box)**	Respondents were asked to type in postcode to validate question.	What is the postcode where you live?	
		(Please type in 4-digit code. *If unsure of postcode*, *please enter digits 9999*)	
$\bar{x}$ = 1.0 ± 0.00			

#### Survey structure and question topics

**Fig 1:**
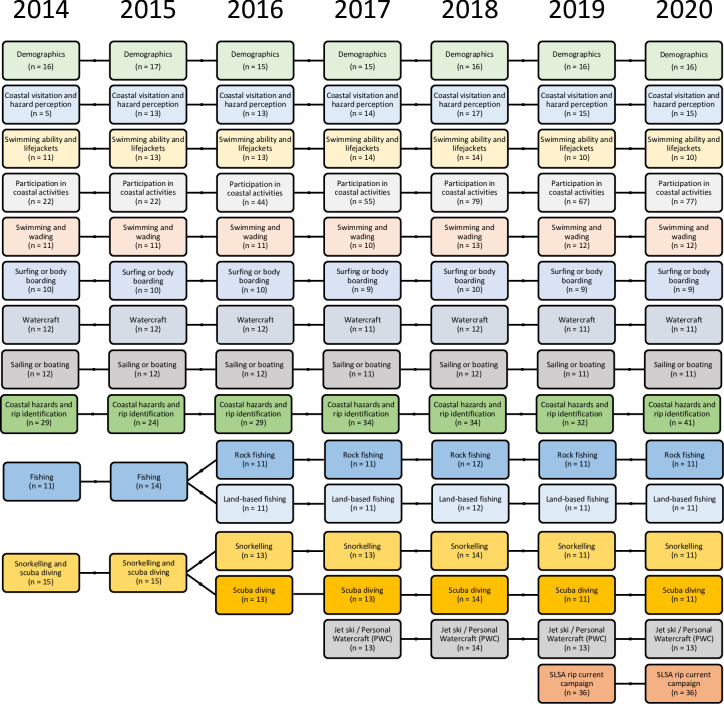
Structure of surf life saving Australia’s national coastal safety survey over time illustrating the number of topics and numbers of questions asked each year. Colours delineate general different topics for each year and illustrate how they have been divided.

The survey structure was designed such that the questionnaire flows from general/broad themes to more specific topics. For example, behaviours and habits are intentionally queried earlier to screen respondents for activity participation and to guide them through the rest of the survey. The survey is structured and delivered using multiple sections within which the main topics are explored. Dominant demographics are asked first to validate respondent eligibility and other demographics (not crucial to the screening process) are asked at the end of the survey, which is standard practise for survey panellists. The survey included 12 sections between 2014–2016, an additional section was added in 2017 on personal watercraft/jet ski participation, and again in 2019 onwards to explore SLSA’s national rip current education campaign (Fig 1). The order of the sections does not change year to year, and the questionnaire always starts with Demographics (Fig 1).

*Topic*: *Demographics*. Demographic related questions are split over two sections (included in one in Fig 1) to maintain engagement and explore age, gender, living arrangements, household composition (i.e. number of adults/children), age of children (5-year brackets), employment status, marital status, highest educational achievement, household income, and ethnicity. Lightspeed ensures data is collected from a variety of postcodes to avoid geographical bias. The number of questions in this topic has varied slightly over the years (Fig 1).

*Topic*: *Visitation*, *hazard perception and skills*. The next topic (split across three sections) asks questions about coastal visitation and participation, hazard perception and skills. Respondents are asked how far away from the coast they live, how often they visit the coast, and what activities they participate in at the coast. From 2015 onwards, this topic was expanded to include questions regarding respondents’ hazard perception of coastal activities. This topic also explores participant’s swimming ability (in general and in the ocean) and their lifejacket usage, which was extended in 2016 to include questions on the type of lifejackets used by respondents.

*Topic*: *General coastal activity participation*. This topic investigates individuals’ general coastal activity participation. Respondents are asked how often and how many hours they normally spend participating in these coastal activities, which location they usually choose for these activities, how hazardous they consider these coastal activities to be, and whether they have ever been rescued or whether they have rescued someone while participating in these activities.

Participation in coastal activities is the largest section of the survey and shows the greatest increase since the survey’s inception (Fig 1). The number of questions within this section rose from 22 in 2014 to 77 in 2020, with a peak of 79 questions asked in 2018. In 2016, questions were added regarding participation in formal organised activities and about the personal motivation for participating in these coastal activities. The single ‘fishing’ category was divided into two categories to be more explicit: ‘rock fishing’ and ‘land-based fishing’; and the single ‘snorkelling/scuba diving’ were separated into two categories reflecting each activity individually (Fig 1). Also, the 2017 survey saw the addition of a new section about ‘jet ski / personal watercraft’ (Fig 1). In 2018, more detailed rescue-related questions were added to gain information on whether people have been rescued or performed a rescue, when and where the rescue happened, whether there were lifeguards or lifesavers patrolling the area and what, if any, flotation device was used. In 2018 and 2020 respondents were asked about the amount of alcohol (i.e. number of drinks) they considered to be reasonable to consume before undertaking coastal activities.

*Topic*: *Specific coastal activity*. This section involves more specific questions in relation to each activity to obtain a more detailed perspective on respondent’s coastal activity participation and behaviour. These questions are activity-specific and are only shown if the respondent indicated they participate in that activity at least three times per year. The specific activities were developed from the most popular coastal activities and include swimming/wading (wading was added in 2015); surfing or body boarding (body boarding was added in 2016); other watercraft (includes kayaks, stand-up paddleboard, canoes); boating or sailing (sailing added 2017); fishing (fishing was separated in 2016 for better clarification into rock fishing and land-based fishing); snorkelling and scuba diving (scuba diving and snorkelling were similarly split in 2016); and the use of personal watercraft (PWC), also known as jet skis was introduced in 2017. There are core questions asked for each activity which remain the same relating to how activity location is chosen and the type and frequency of safety equipment or and safety practices used; and some relevant activity-specific questions were added when needed. Each activity section has between 10 and 25 questions.

**Fig 2:**
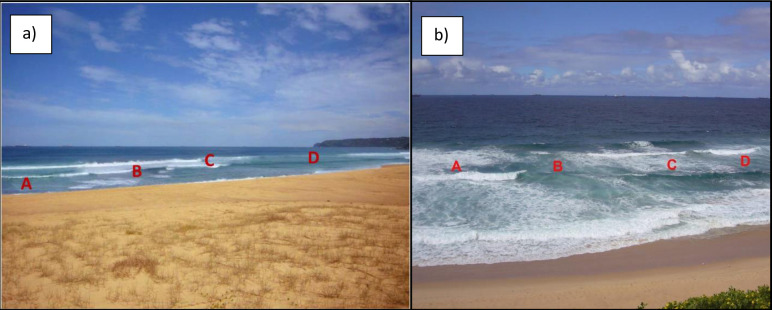
Pictures in the surf life saving Australia national coastal safety survey shown with each question asking ‘Please look at the picture below and identify the location of rip currents, if any. *(Select all that apply*)’ to which the responses are coded as (0) No rip currents in this picture, (1-4) Location A through to Location D, and (99) can’t say.

*Topic*: *Coastal hazards and rip currents*. All participants are asked about their understanding and perception of coastal hazards and rip current identification. To measure real and perceived rip current knowledge, the NCSS displays two images of rip currents to each participant, one image has been used every year since 2014 (Fig 2A) and the other every year since 2015 (Fig 2B). The participants asked to identify the rip current location in each photo or indicate its absence. Respondents are also asked about strategies that can be used to escape a rip current. Other questions in this section relate to coastal safety information and coastal safety authorities. In 2020, nine questions were added specifically on the respondent’s experience with marine stingers (i.e. jellyfish).

*Topic*: *Annual focus areas*. Each year also contains questions relating to a specific focus area such as alcohol consumption and activity participation (2018 and 2020), bystander rescue experience (experience in performing rescues and being rescued (2017–18), marine stingers (2020), and SLSA’s rip current campaign (2019–20).

#### Mapping survey questions and charting changes

To chart the major themes and track evolution of the survey, a *question mapping* process was conducted. The exact text for all questions from each year of the survey, and their response options, were collated into a spreadsheet (Microsoft® Excel®) and marked according to the theme of the question: demographics, hazard perception, activity participation, rip currents, safety practices, special features, swimming ability and visitation. This thematic charting differs from the topics and sections identified above; each topic may contain questions of several different themes. For example, the topic section on surfing would contain questions on hazard perception, activity participation, and safety practices. Additionally, the question map added each subcomponent of multi-response questions as individual questions. For example, question G4 (Section G, question 4) in 2020 asked about safety practices followed while surfing. The question contained multiple sub-components and since each required an individual answer was therefore entered as six separate questions (G4a-G4f) into the question map (Table 2). Consequently, the total number of questions from the questionnaire and the question map differ considerably; between 2014 and 2020, 690 questions were asked in the questionnaire, which, including all their subcomponents, actually represents 1,592 separate questions. The following descriptions of the survey are derived from the *question map process*, which treats each subcomponent of a question as its own inquiry.

**Table 2:** Example question (G4) from 2020 survey.

G4 Main question:					
*Please indicate how often you personally follow each of these practises when you go surfing or body boarding*? *(One answer per row)*					
Subcomponent and text	Answer options (choose one)				
A: Surf with at least one other person you know	Always	Most of the time	Sometimes	Never	Can’t say
B: Check surf conditions with a lifesaver, lifeguard or other authoritative source	Always	Most of the time	Sometimes	Never	Can’t say
C: Surf in conditions that are appropriate for your skill level	Always	Most of the time	Sometimes	Never	Can’t say
D: Use safety equipment such as leg ropes, safety fines, helmets or buoyancy aids	Always	Most of the time	Sometimes	Never	Can’t say
E: Avoid surfing under the influence of alcohol / drugs	Always	Most of the time	Sometimes	Never	Can’t say
F: Follow the advice of local lifesaver or lifeguard when you are on a patrolled beach	Always	Most of the time	Sometimes	Never	Can’t say

**Fig 3:**
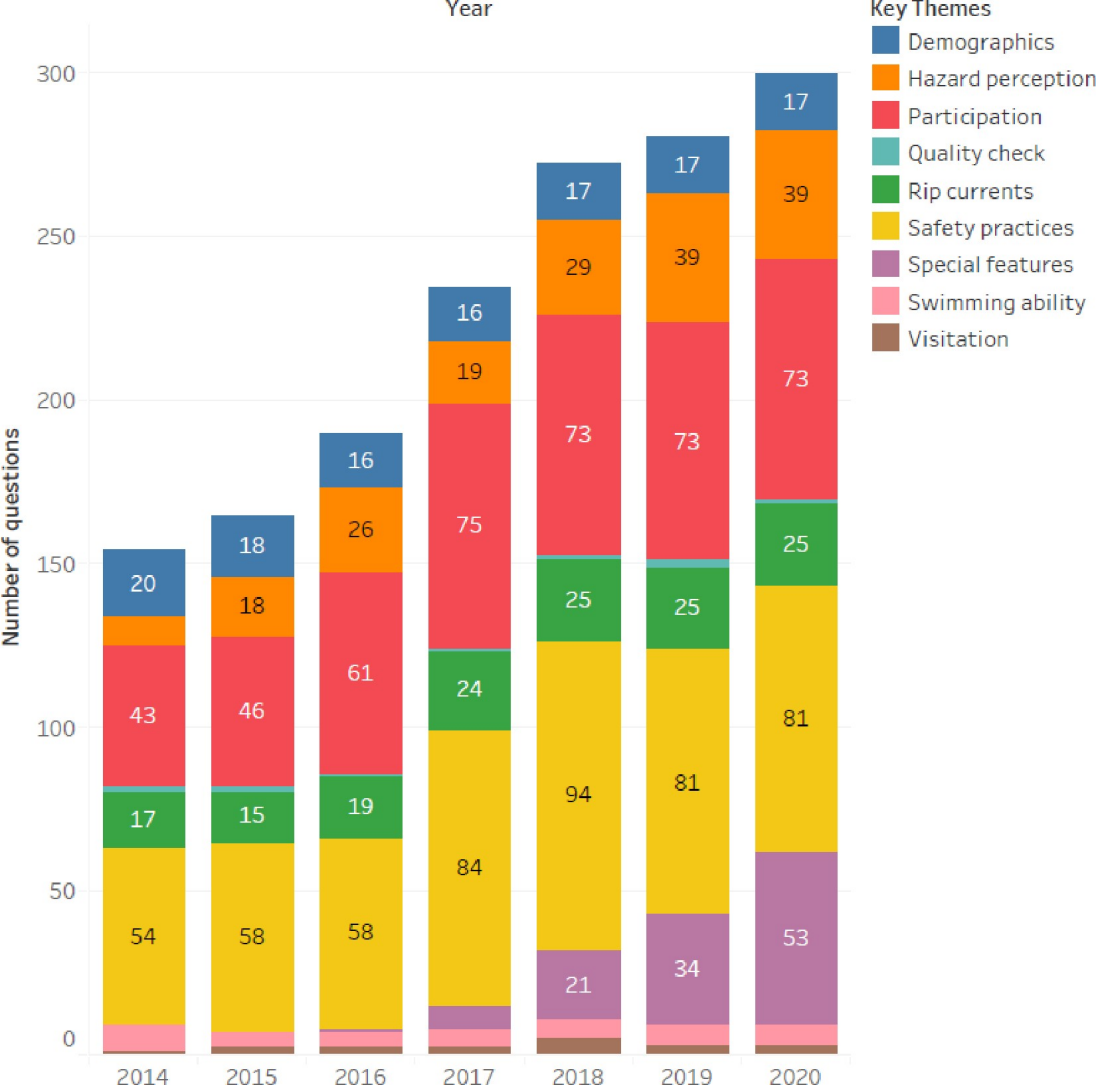
Structural proportions of the surf life saving Australia national coastal safety survey illustrating the themes and number of questions within each survey. NB: Not all respondents are required or eligible to answer all questions, and while the number of questions have doubled since 2014, the survey length (minutes) has only increased by 40% (4.5 minutes).

The total number of questions increased steadily over the seven years, from 154 possible questions in 2014 to 299 possible questions in 2020, highlighting the size and the scope of the National Coastal Safety Survey (Fig 3). Safety practices and activity participation questions account for the majority of questions (52%-68%) in each year of the survey, but are only asked to specific groups of respondents and therefore represent a small proportion of the survey length. Demographics, hazard perception and rip current questions account for 6% to 14% each of the total survey. Swimming ability and coastal visitation questions represent the smallest proportions of question asked comprising 2–5% and 1–2% respectively.

The largest proportion of the survey explores safety practices, totalling 510 questions over the years (32%; Fig 3). In addition to standalone questions on lifejacket usage and coastal safety information sources, respondents are asked a series of safety questions corresponding to each coastal activity they participate in. A significant increase in questions occurred in 2016 (from 58 to 84) due to the separation of the fishing and scuba/snorkelling sections previously described, and again in 2017 with the addition of a new section on personal watercraft / jet ski (Fig 1). In 2018 only, questions were added regarding how the use and frequency of personal safety practices have changed compared to five years prior. It should be noted here that while the number of questions has theoretically doubled, not all respondents are eligible to answer all questions. The average completion length of the survey began at 14 minutes in 2014, grew to 16 minutes in 2017 and was 19.5 minutes in 2020. While the number of available questions has doubled, the survey length (length of interview) has not since survey respondents only complete sections relevant to their participation.

Coastal ‘activity’ participation questions comprise the second largest theme equalling 444 questions from 2014–2020 (Fig 3). For each activity, respondents are asked which coastal environment they usually go to, what criteria are important in location choice, how often and how much time they spend participating in these activities. Between 2015–17, questions were asked whether respondents participated in formal organised activities (e.g. swimming or dive clubs). Participation questions also include inquiries about whether the respondent has ever been rescued while participating in each coastal activity. As previously described, an increase is observed in 2016 relating to the division of fishing and scuba/snorkel activities, and the addition of personal watercraft / jet ski activities in 2017 (Figs 1 and 3). From 2018, respondents were also asked to assess their own level of expertise and experience in coastal activities.

The number of questions on demographics remained relatively constant (16–20 questions; Fig 3) and was expanded in 2015 to include a question on the respondents’ ethnic background. A small number of questions ask respondents about their general swimming ability, their swimming ability in the ocean and whether they have ever participated in formal swimming lessons (Fig 3). Hazard perception questions for each activity, the coastal environment, and dominant coastal hazards (i.e. rip currents, waves, sharks and sun exposure) has increased from nine questions in 2014 to 39 questions in 2020 (Fig 3) and included respondents’ self-assessments of their ability to take risks (in 2016, 2019 and 2020). Questions on rip currents varied (15–25 questions), increasing between 2015–2017 (Fig 3). Only in 2017 were participants asked to answer ‘*true’* or ‘*false’* against statements related to rip currents, e.g. ‘*only tourists get caught in rips’ or ‘rips only take lives of poor swimmers’*.

*Survey evolution*: *Year to year changes*. The majority of questions in the survey were asked to eligible respondents each year, maintaining the same structure and wording. Nevertheless, there were some notable minor and major changes over time. Minor changes are those which resulted in no change to the question meaning, but involved slight changes to sentence structure, or the addition of clarifying language or extra response categories. Examples include when questions related to the activity ‘swimming’ were updated to ‘swimming or wading’ (since many people enter the water at the coast but do not technically ‘swim’); or when response categories to questions related to safety practices added ‘shark deterrent devices’ as an option for safety equipment.

Major changes are those which might have altered the interpretation of the question and resulted in a different response. For example, before 2017 respondents were asked if they had ever been caught in a rip current. While the aim of this questions was to determine how many people had been caught unintentionally, the wording did not specify this. As a result, surfers who often use rip currents to help them get beyond the breaking waves also selected ‘*yes*’, which skewed responses. Therefore, the addition of the word ‘unintentionally’ in 2017 impacted the interpretation of the question and subsequent responses.

The most significant change to the survey structure was when activity categories were divided in 2016 from a single ‘fishing’ category into separate ‘rock fishing’ and ‘land-based fishing’ categories, and a single ‘scuba diving/snorkelling’ category to two activity separated categories, as previously described. While these changes increased the total number of questions and changed the overall structure of the survey, it significantly helped to improve understanding of the respondents’ activity participation and safety practices. Another example of a major change is when question type changed (e.g. from open text to categorical question), which meant that data from these two question types cannot be combined for analyses.

Another significant change which impacted responses was the extension of the target population for 16–69 years to 16 years of age and above in 2018. This made the survey results more representative of the population and facilitates a more accurate understanding of coastal visitation, especially since drowning rates are consistently higher for older adults [5, 36–38]. This change was made for practical reasons, as prior to 2018, it was understood that internet penetration was relatively poor in older Australians (70+ years of age) and would not reflect the actual population, but now is considered to be appropriate for accurate data collection for Australians aged 35 years and older [34].

### Statistical analyses

Crude fatal coastal drowning rates were calculated for those aged 16 years and older overall and by sex, age, and activity. Fatal coastal drowning statistics were derived from Surf Life Saving Australia’s data which is primarily sourced from National Coronial Information System (NCIS) [13]. In order to accurately calculate rates, drowning deaths of non-residents were excluded from the analyses. Resident population data were sourced from the Australian Bureau of Statistics. To calculate rates, a 3-financial year average of the deaths (2017/18-2019/20) was used as the numerator and a 3-year average of the population (as at June each year, i.e., June 2018-June 2020) as the denominator.

In order to revise crude fatal drowning rates based on exposure, the denominator (population) was revised based on the average proportion of respondents across three years of the survey who stated they had visited the coast at least three times per year. Revised rates were then calculated using the 3-year average of deaths as the numerator and the exposed population as the denominator. Relative risk (RR) with a 95% confidence interval (CI) was calculated comparing drowning risk among the total population to the revised drowning risk for the exposed population.

### Ethics

This survey has been conducted within approved market research procedures, and the secondary use of the data collected in this survey in academic research has been approved by UNSW Sydney Human Research Ethics Committee Panel B: Arts, Humanities & Law (HC200950; approved 16^th^ February 2021). This HREC is constituted and operates in accordance with the National Health and Medical Research Council’s (NH&MRC) National Statement on Ethical Conduct in Human Research (2007). The processes used by this HREC to review multi-centre research proposals have been certified by the NH&MRC.

## Results

In order to achieve a representative sample of the Australian population, across the seven years of the survey, data for a total of 10,567 respondents’ has been collated. Numbers of respondents are evenly spread across the years and between males and females and age groups (Table 3). The activity category of swimming and wading was the most popular coastal activity reported by respondents each year followed by boating, snorkelling, surfing and other watercraft (Table 3).

**Table 3:** Number and proportion of survey respondents each year by key demographics and activities.

Variable	2014	%	2015	%	2016	%	2017	%	2018	%	2019	%	2020	%
Total respondents	1389	100.0	1463	100.0	1431	100.0	1458	100.0	1597	100.0	1642	100.0	1587	100.0
**Gender**														
Male	695	50.0	732	47.5	716	50.0	729	50.0	780	48.8	802	48.8	775	48.8
Female	694	50.0	731	47.4	715	50.0	729	50.0	817	51.2	840	51.2	812	51.2
Age group *														
16–34 years	532	38.3	562	38.4	549	38.4	559	38.3	517	32.4	532	32.4	514	32.4
35–54 years	519	37.4	554	37.9	525	36.7	514	35.3	492	30.8	495	30.1	507	31.9
55+ years	338	24.3	347	23.7	357	24.9	386	26.5	588	36.8	615	37.5	566	35.7
Activity **														
Swimming/Wading	701	50.5	720	49.2	844	59.0	904	62.0	858	53.7	817	49.8	828	52.2
Boating	297	21.4	266	18.2	258	18.0	301	20.6	210	13.1	234	14.3	215	13.5
Jet ski/PWC***	N/A	0.0	N/A	0.0	N/A	0.0	64	4.4	36	2.3	47	2.9	61	3.9
Land-based fishing	N/A	0.0	N/A	0.0	297	20.4	274	18.8	252	15.8	253	15.4	212	13.4
Rock Fishing	N/A	0.0	N/A	0.0	107	7.5	95	6.5	100	6.3	100	6.1	72	4.5
Watercraft	129	9.3	126	8.6	134	9.4	166	11.4	105	6.6	102	6.2	106	6.7
Surfing	180	13.0	154	10.5	167	11.7	152	10.4	130	8.1	122	7.4	114	7.2
Snorkelling	N/A	0.0	N/A	0.0	193	13.5	214	14.7	168	10.5	152	9.3	177	11.2
Scuba Diving	N/A	0.0	N/A	0.0	41	2.9	31	2.1	32	2.0	53	3.2	35	2.2

*Note: 2014–17 respondents were aged 16–69, 2018 onwards was 16+

**Note: proportions of respondents by activity will not total 100% as respondents may participate in more than one activity.

*** PWC = Personal Watercraft

**Fig 4:**
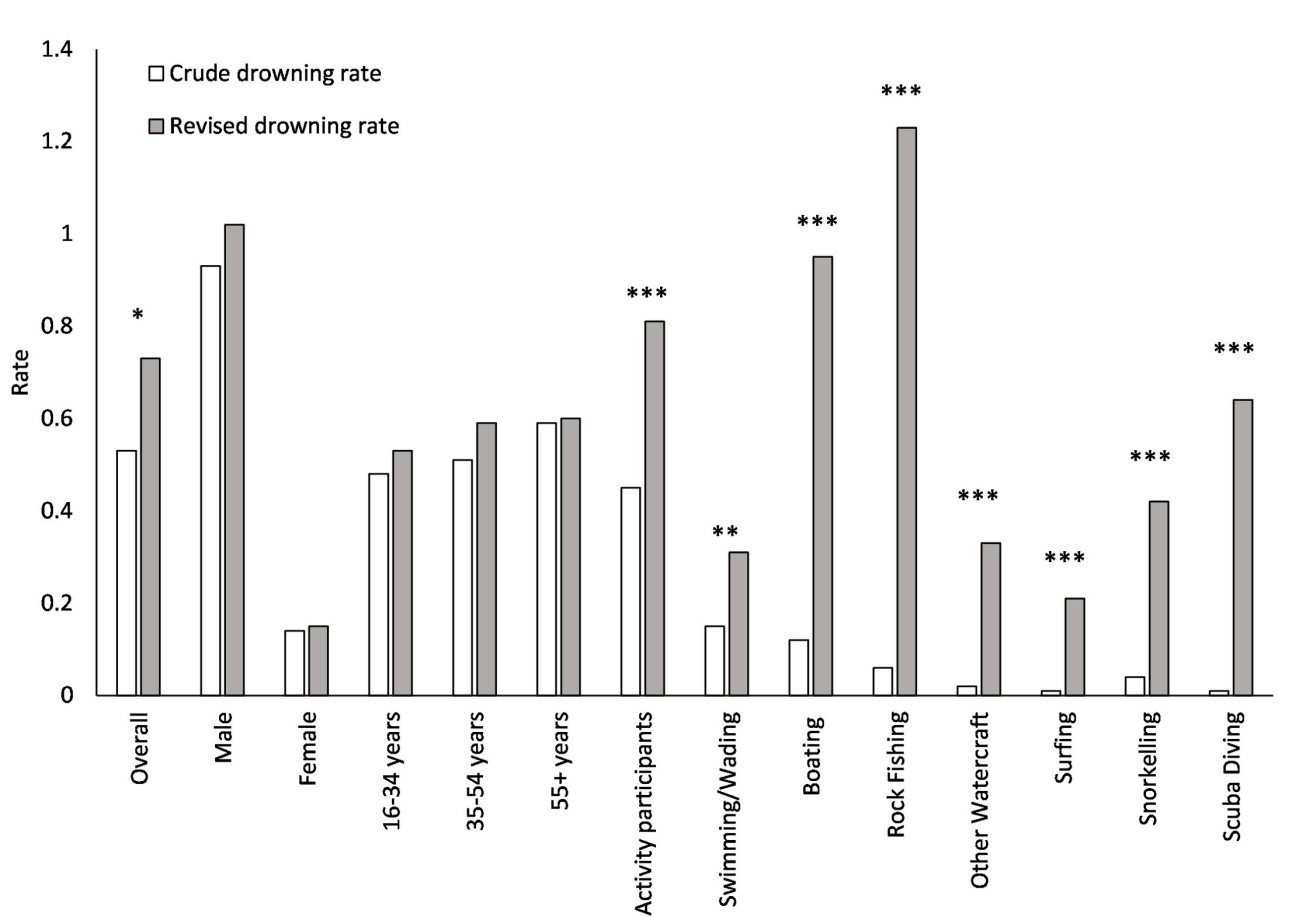
Crude drowning rate per 100,000 population compared with the revised drowning rate per 100,000 participants calculated using participation data collected by the National coastal safety survey. Levels of significant differences between the rates are illustrated with asterisks: p<0.02 (*), p<0.001 (**), p≤0.0001(***), and highlight target groups for which this methodology is important.

Preliminary analyses highlight the differences in fatal drowning rates when exposure is considered (Fig 4, Table 4). The most pronounced differences occur when participation in coastal activities is considered (Fig 4, Table 4). Fatal drowning rates for rock fishing vary from 0.06 per 100,000 resident population, to 1.23 per 100,000 exposed population (RR = 19.29; 95%CI: 8.94–41.61; p<0.0001), the highest fatal drowning risk of any activity. Similarly, the rate of fatal drowning during boating increased from 0.12 per 100,000 resident population to 0.95 per 100,000 people exposed (RR = 8.01; 95%CI: 4.55–14.10;; p<0.0001), and scuba diving from a rate of 0.01 to 0.64 per 100,000 exposed (RR = 43.43; 95%CI: 8.77–215.18; p<0.0001) (Table 4).

**Table 4:** Preliminary estimates of fatal unintentional drowning rates for dominant coastal activities, with and without exposure using data collated for decedents aged 16 years and above (calculated for reporting periods 2017/18FY-2019/20FY). Bold type indicates significance.

	3-year average drowning deaths (16+)	3-year average population (16+)	Crude drowning rate/100,000 pop.	% of those who visit the coast at least three times per year (16+)	Revised population exposed/ 100,000 pop.	Revised rate considering exposure	Relative risk (95% confidence interval; p-value) comparing crude population drowning risk to exposed population drowning risk
Total	107	20318916	0.53	71.7	14566667	**0.73**	**1.39 (1.07–1.82); p = 0.0149**
**Gender**							
Male	93	9985395	0.93	91.5	9136000	1.02	1.09 (0.82–1.46); p = 0.5444
Female	14	10333521	0.14	92.7	9576000	0.15	1.08 (0.51–2.26); p = 0.8404
**Age group**							
16–34 years	32	6727102	0.48	90.1	6062000	0.53	1.11 (0.68–1.81); p = 0.6771
35–54 years	34	6602859	0.51	87.8	5796544	0.59	1.14 (0.71–1.83); p = 0.5913
55+ years	41	6988955	0.59	98.1	6853456	0.60	1.02 (0.66–1.57); p = 0.7969
**Activity**							
Total coastal activity participants	92	20318916	0.45	55.8	11333333	**0.81**	**1.79 (1.34–2.39); p = 0.0001**
Swimming/Wading	30	20318916	0.15	47.7	9691224	**0.31**	**2.10 (1.26–3.48); p = 0.0041**
Boating	24	20318916	0.12	12.5	2537977	**0.95**	**8.01 (4.55–14.10); p < 0.0001**
Rock Fishing	13	20318916	0.06	5.2	1053434	**1.23**	**19.29 (8.94–41.61); p < 0.0001**
Other Watercraft	4	20318916	0.02	6.0	1212085	**0.33**	**16.76 (4.19–67.03); p < 0.0001**
Surfing	3	20318916	0.01	7.0	1423953	**0.21**	**14.27 (2.88–70.70); p < 0.0001**
Snorkelling	8	20318916	0.04	9.4	1915340	**0.42**	**10.61 (3.98–28.27); p < 0.0001**
Scuba Diving	3	20318916	0.01	2.3	467851	**0.64**	**43.43 (8.77–215.18); p < 0.0001**

NB: Land-based fishing and Personal Watercraft (e.g. jet skis) have been excluded from these exploratory analyses due to low numbers of drowning deaths recorded for each activity.

Survey responses are used each year in SLSA’s research outputs such as the National Coastal Safety Reports [39] and Coastal Safety Briefs [27, 40, 41]. Annual survey data will be collated to build a comprehensive dataset that can be used for longitudinal studies and combined to create a larger sample size for questions that span multiple years. The data maintained in this master dataset will be used to develop participant ‘populations’ for specific demographics that will become the denominator in calculating exposure rates for research pertaining to coastal-related morbidity and mortality in Australia, a topic that is poorly represented in drowning prevention literature to-date [18].

## Discussion

This research methodology paper outlines the process of developing and conducting a nationally representative coastal safety survey which aims to identify at-risk groups and better inform drowning prevention campaigns and education. Our results also highlight the need to consider the actual exposed population when calculating accurate mortality rates, especially for individual activities. The challenges encountered during this process highlight considerations that may be necessary when developing similar survey tools.

The unique dataset developed using this method provides an ongoing comprehensive study of coastal visitation, participation, knowledge, behaviour and attitudes across Australia. It facilitates the calculation of more accurate fatal coastal drowning rates per 100,000 head of exposed population, as demonstrated in the preliminary analyses presented in this paper which shows risk of drowning is up to 43 times higher than originally calculated among scuba divers and 19 times higher among rock fishers in addition to other findings. More refined understanding of the impact of exposure on drowning risk, informs the identification of at-risk groups for drowning prevention interventions. This has already been seen in the enhanced advocacy, education, and legislation improvements (with associated enforcement) aimed at preventing drowning and aquatic injury among rock fishers [42]. Findings generated by this study will inform future refinement of preventive approaches based on risk.

It is the only current dataset of its type in Australia, with the next most systematic data collection being occasional sub-national level rip current studies [10, 11] or past broader surveys into sport and physical recreation [43, 44], though these are not collecting ongoing data. This dataset, built using the described survey methodology, will allow for deeper analysis into a range of topics. This includes the identification of at-risk groups, such as those with self-reported i) poor skills (i.e. swimming skills) [45]; ii) lack of safety knowledge (i.e. unable to spot a rip) [12]; and iii) risk-taking behaviours (i.e. swimming at unpatrolled locations [10, 11], not wearing a lifejacket [46] or engaging in aquatic activity under the influence of alcohol [13].

Future research using this dataset will better inform prevention and mitigation approaches to address coastal safety. Improved denominator data will allow for the revision of coastal drowning and injury rates thus enabling better identification of at-risk groups to inform coastal drowning and injury prevention strategies including public awareness and education campaigns. The longitudinal nature of this data will also facilitate evaluations of the impact of drowning prevention interventions on knowledge, attitudes and behaviours.

Across the multiple years of the survey, important lessons have been learned. Changes to survey questions have been made as the questionnaire evolved. While this strengthens comprehension and thus the quality of the data collected, any changes made limits comparability with previous iterations of the survey. However, refinement of the questionnaire has been necessary at times, for example, when terminology used in the questionnaire did not have the intended meaning among respondents. One such example, was people who ‘fish from rocks’ but did not respond that they participated in ‘rock fishing’. This cognitive dissonance between respondents practice requires further investigation. As such, the survey tool changed the term ‘rock fishing’ to be ‘fishing from rocks’, which more accurately captured the number of fishers who fish from rocky areas, also known as rock fishers. Another pertinent example is the aforementioned addition of the word ‘unintentionally’ to the question ‘have you ever been caught in a rip’ to exclude surfers who intentionally enter rip currents. This highlights the importance of identifying conflicts and incorporating the right language to get a usable and meaningful response. Similarly, there are three different time references used in the survey questions, e.g. participation in the last 12 months, participation ever, and recent participation experiences (unspecified time). These question-specific time frames need to be considered when analysing, comparing, and interpreting the data. Moreover, consideration of the time of year that the survey is administered, April–Autumn in Australia, is required during analysis. These examples demonstrate the commitment to evaluation and continual improvement of the survey as a tool, an additional strength of the project.

There are limitations, however, regarding small cohorts of respondents and extrapolating those data to the general Australian population through the representative sample. One example is the estimation around the proportion of the population who participate in scuba diving and snorkelling. The extrapolated numbers of scuba divers from this study have, since scuba diving was separated into its own category in 2016, been calculated from a base of approximately 45 participants each year. These extrapolated numbers of participants differ from other research [25] where risk estimates calculated using denominators developed from other sources are more conservative. While the extrapolation process incorporated in this survey suits the intended broader scope of this study, for activities where participation levels are lower, this approach may be limited and interpretations need to be considered with caution. For such instances, exploration of alternative approaches, e.g. where the number of survey respondents are increased or the focus of the survey is more targeted and detailed, are recommended to determine the most effective and accurate results.

There are many strengths associated with this study, including its representative sample generating a unique and longitudinal dataset which provides data across a range of topics to guide efforts to reduce drowning and promote safety at coastal locations in Australia. There are, however, limitations which may provide opportunities for further research. One such example is the online accessibility of the survey and challenges around preventing respondents from concurrently searching for answers to knowledge questions (i.e. how do you define a rip?) [47]. Similarly, it must also be acknowledged that past experiences, other experiences, social influences, socially dictated attitudes and behaviours play a role in an individual’s identity and may influence thoughts and behaviours. The survey has not been built to delve into respondent psychology but to build an understanding of opinions, perceptions and behaviours regarding specific coastal safety questions. OmniPoll Market Research, as a research agency, must ensure that the questions are understood by all, and that the general public can provide an answer. Specifically, the survey questions must be relevant, clear (not vague), concise, purposeful, guiding but not leading, and unequivocal (single-dimensional). The questions in this survey have been written such that they are almost all about the respondent, with the inclusion of a limited number of questions which are reported by the respondent for the entire household (e.g., household income and composition, or whether there are lifejacket home) as is common market research practice. The respondent is asked to answer based on their own experience, but there are no controls as of yet to test and account for external influences.

Another limitation in some regards relates to survey responses being de-identified, to maintain anonymity, but thereby does not allow for individual follow-up to identify changes among respondents and measure changes in their responses over time, or conversely mitigate against multiple surveys being completed. For the latter concern, the cohort database available to participate in the survey turns over 50% of potential respondents per year and Lightspeed prevents respondents from participating in the survey in the following year, this may result in a small proportion of the respondents responding to the survey in alternate years. While the questionnaire is designed to minimise social desirability bias, it remains possible for respondents to be influenced by social desirability bias or demand characteristic bias [35, 48] and respond in a manner that would differ to someone who was unfamiliar with the survey.

The population distribution in Australia also poses challenges for securing a truly representative sample. For example, places with smaller populations, e.g. the Northern Territory (Australia’s least populated governing body at this level) may have the same concerns highlighted in the above example about small cohorts. Similarly, despite high internet penetrance [49], the online nature of this survey may exclude some lower socio-economic households without reliable internet access. Similarly, the online nature of the survey may also exclude groups (such as the elderly) with lower electronic and telecommunications literacy. While such disadvantages need to be acknowledged, they have not been found to outweigh the advantages of online data collection [50].

Other limitations that may need to be considered for future surveys involve the need for more detail about the respondents themselves (although the authors acknowledge that significant changes to the current survey format may incur extra costs and may reduce completion rates). For example, categorising respondent genders to male and female excludes the component of the Australian population who are gender diverse, although data regarding this population is currently not reliable [51]. The current questionnaire also does not currently ask questions for migrants or culturally and linguistically diverse (CaLD) respondents such as main language spoken at home and how long the respondent has lived in Australia. While people born in Australia do drown in higher numbers, there is a significant risk of drowning among migrants [52, 53], this must be an important consideration in future iterations of the questionnaire. The survey captures the responses of Australian residents only, but not of those of international visitors. Alternative data collection methods are required to identify the coastal safety behaviours, knowledge and attitudes of international tourists to Australia, a group known to be at risk of coastal drowning [54]. Similarly, the questionnaire is quality checked and tested for comprehension using a small sample, but it has not been deliberately tested among respondents for whom English is a second language. Finally, given the national (broad) scope of this survey, it has been designed specifically to reach a larger sample with a relatively short survey (length of interview should rarely exceed 15 minutes), the survey is therefore unlikely to ensure a complete array of all potential dimensions. This is a common strategy, however, to avoid respondent fatigue with longer surveys and maintain a high completion rate. These current gaps are all worthy of consideration as means of strengthening the survey in the future.

## Conclusions

Coastal drowning is a significant contributor to the overall drowning burden, globally, as well as in Australia. The preliminary results from this paper demonstrate the importance of accurately estimating exposed populations, with the risk of drowning changing dramatically, especially for specific activities. More detailed information on the skills, behaviours, knowledge, and attitudes of the coastal activity participants is vital to understand exposure to risk and to develop targeted strategies more likely to improve safety and reduce drowning. Using this tool to enhance identification of populations and individuals at greater risk of drowning or coastal injury helps to shape effectual prevention interventions for those who need them most. This methods paper outlines the approach and lessons learned in the development and conduct of a nationally representative survey of the Australian population about coastal safety. Such data is largely lacking in the published literature identifying this as a knowledge gap which urgently needs to be addressed. Analyses of the unique dataset will inform research that will underpin development and evaluation of coastal drowning prevention initiatives. It is hoped that other countries with a similar coastal drowning burden may look to this study to develop similar data collection tools in their own countries.
